# Successful treatment of multidrug-resistant *Pseudomonas* a*eruginosa* keratitis with meropenem eye drops — a case report

**DOI:** 10.1186/s12348-023-00363-0

**Published:** 2023-09-16

**Authors:** Carolin Elhardt, Armin Wolf, Christian Maximilian Wertheimer

**Affiliations:** Department of Ophthalmology, University Hospital Ulm, Prittwitzstrasse 43, 89075 Ulm, Germany

**Keywords:** Pseudomonas aeruginosa, Keratitis, Meropenem, Multi-resistance, Antibiotics, Case report

## Abstract

**Background:**

This case report describes the course and therapeutic management of a fast-spreading bacterial keratitis caused by multidrug-resistant (MDR) *Pseudomonas aeruginosa (P. aeruginosa)*.

**Case presentation:**

A 27-year-old male contact lens wearer presented with a multi-resistant, fast spreading *P. aeruginosa* keratitis. After initial resistance to various antibiotic therapies, testing revealed a MDR *P. aeruginosa*. The keratitis was treated successfully with specially prepared 50 mg/ml off-label meropenem eye drops for 18 days as well as systemic meropenem for seven days with rapid improvement of the corneal infiltrate.

**Conclusion:**

This case report demonstrates the combination of topical and systemic meropenem as a useful treatment option for corneal ulcers caused by MDR *P. aeruginosa*.

## Background

*Pseudomonas aeruginosa* (P. aeruginosa) is a gram-negative, opportunistic pathogen. Due to its diverse metabolic pathways and large repertoire of pathogenic mechanisms, *P. aeruginosa *is able to adapt to a wide variety of environments, causing multiple resistances [1]. Despite various protective mechanisms of the eye, it is one of the most common pathogens causing vision-threatening eye-infections. Risk factors for a *Pseudomonas *keratitis are particularly high in pre-existing contact lens wearers, as well as patients with ocular surface disease, ocular trauma, and those who have undergone ocular surgery [2]. It is the most identified causative organism in contact lens-related keratitis [2].

Resistance of gram-negative bacteria to several groups of antibiotics can result in so-called multidrug-resistant gram negative bacteria (MRGN) [3]. According to a report on antimicrobial resistance by the European Centre for Disease Prevention and Control, 3.9% of *P. aeruginosa* were resistant to three (3MRGN), 2.8% to four (4MRGN) and 3.4% to five antimicrobial groups. A previous review reported that the resistance of *P. aeruginosa* depends significantly on the year and location in the world. The range of resistance to ciprofloxacin is 0–30% (to gentamicin 0–46%, and to 3^rd^ generation cephalosporin 0–14%).

The potential ineffectiveness of standard therapies poses a challenge for the rapid and successful treatment of multidrug-resistant (MDR) organisms. This case report demonstrates an effective treatment of a MDR *P. aeruginosa* with a combination of topical and systemic off-label meropenem.

## Case presentation

**Fig. 1:**
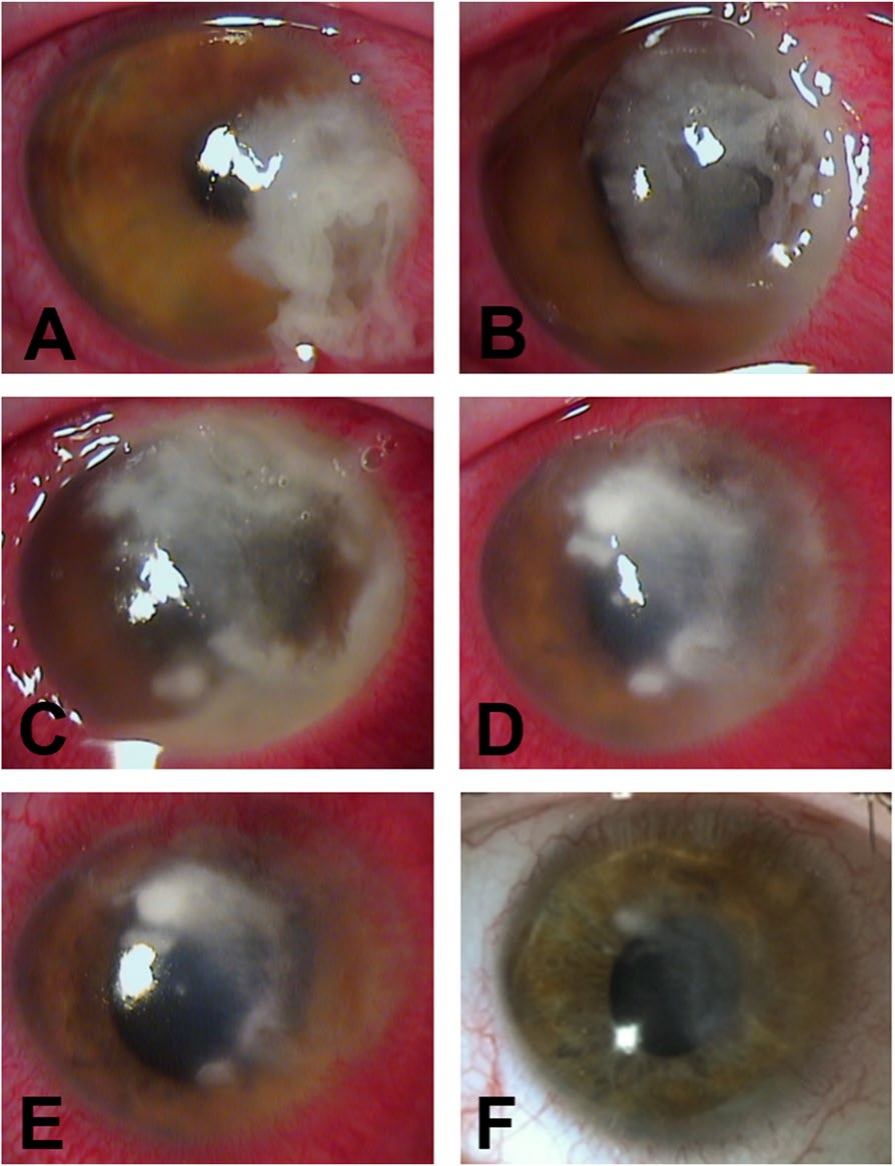
Photographic images of anterior eye segment of the right eye: **A**) Day 1; **B**) Day 3; **C**) Day 7; **D**) Day 15; **E**) Day 20; **F**) after 4 months

**Fig. 2:**
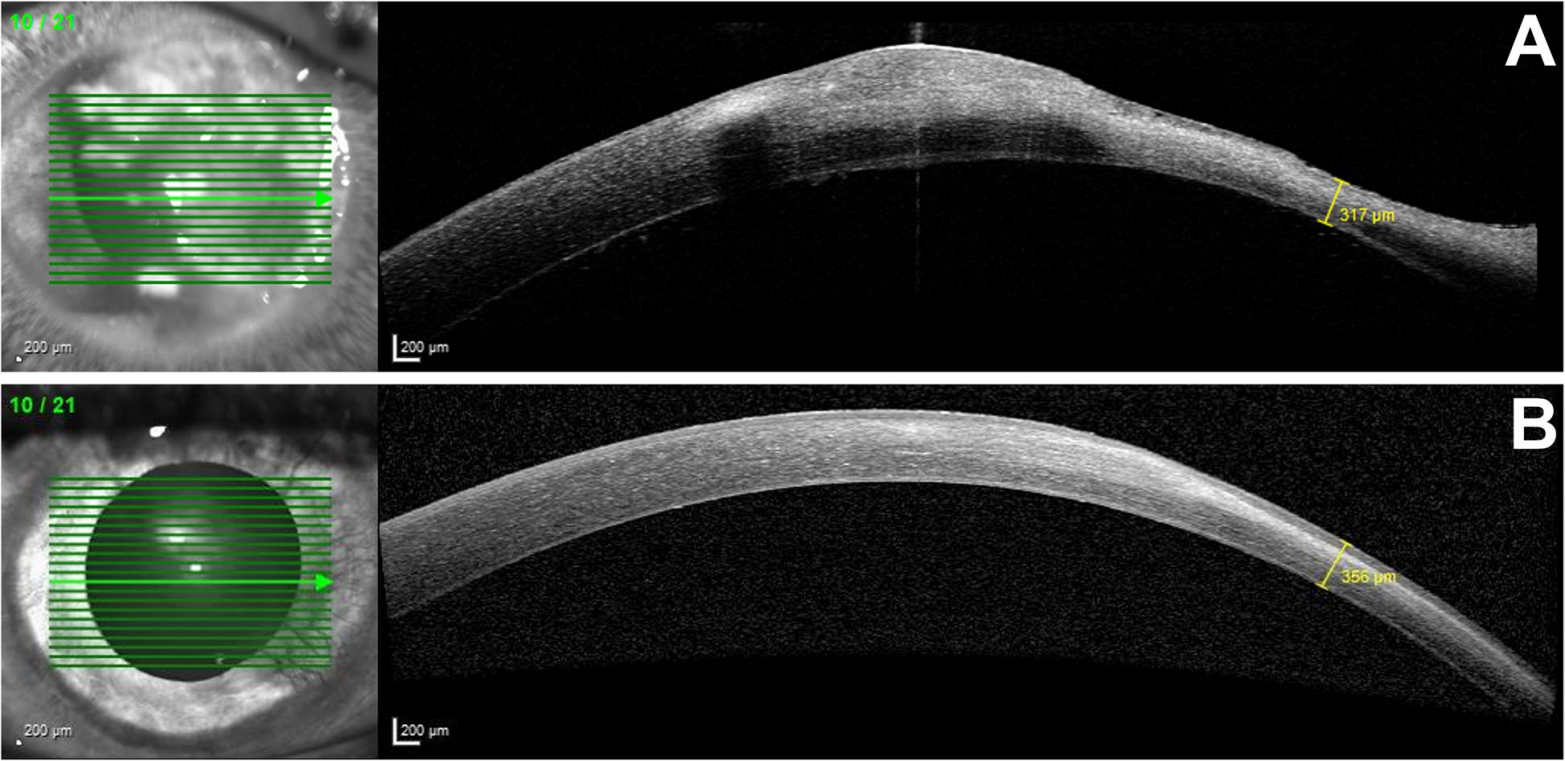
Anterior segment optical coherence tomography of the right eye: **A**) Day 8, thinnest corneal point 317 µm; **B**) after 8 months, corneal thickness 356 µm

A 27-year-old male contact lens wearer was referred to our outpatient ophthalmology clinic for the first time by his primary care ophthalmologist with an urgent case of keratitis of presumed bacterial origin of the right eye. Symptoms started four days before the referral. Treatment with a combination eyedrop containing gentamicin and dexamethasone was prescribed by the patient’s primary care ophthalmologist. The patient started taking the eye drops 4–6 times a day before referral. The last documented corrected visual acuity prior to the onset of the keratitis was 1.0. His initial visual acuity at presentation with his own glasses was 0.1 (1.00 logMAR) in the affected right eye and 1.0 (0.00 logMAR) in the unaffected left eye. Ophthalmic examination revealed a mucopurulent infiltrate in the cornea approximately the size of 25% of the cornea involving the limbal region (Fig. 1A). Stromal melting had already occurred and the corneal thickness at the thinnest point was 320 µm (Fig. 2A). In summary, based on the history and appearance, the first clinical suspected diagnosis was a corneal ulcer caused by *P. aeruginosa*; other bacteria or fungi were also considered for differential diagnosis. Due to the severity and size of the findings, the patient was admitted to inpatient care. Furthermore, a corneal ulcer scraping, a conjunctival swab, and the contact lens case were sent to microbiology for direct staining, culture, and antimicrobial susceptibility testing. A combination preparation eyedrop of polymyxin-B, neomycin, and gramicidin as well as moxifloxacin eye drops were started and administered by the nurse on an hourly alternating basis at day and night. In literature, the combination of polymyxin-B, neomycin and gramicidin [4] as well as moxifloxacin [5] have been described as highly effective for the treatment of bacterial corneal ulcers. With this therapy, we used commercially available antibiotic eye drops with good clinical effectiveness, a broad-spectrum antibacterial activity, and few side effects. In addition, atropine eye drops 0.5% were used throughout the inpatient stay (all eye drops including concentrations are shown in Table 1).

**Table 1:** Concentration of administered topical eyedrops

Eye drop	Concentration
Polymyxin-B	7500 I.U. / ml
Neomycin	3500 I.U. / ml
Gramicidin	0.02 mg / ml
Moxifloxacin	5 mg / ml
Atropine	0,5%
Gentamicin	5 mg / ml
Voriconazole	1%
Meropenem	50 mg / ml
Levofloxacin	5 mg / ml

The next day, a rapid enlargement occurred, so gentamicin eye drops were added. These eye drops have a good spectrum of efficacy in the gram-negative area.

On the third day, the size of the infiltrate increased to approximately twice its initial size (Fig. 1B). The differential diagnosis of a possible fungal infection was covered with hourly voriconazole 1% eye drops.

On the sixth day, there was a further marked worsening of the findings with new hypopyon formation. Topical levofloxacin was started and administered every hour alternating with gentamicin instead of moxifloxacin and the combination preparation of polymyxin-B, gramicidin, and neomycin.

A microbial detection with antibiogram (Table 2) of the corneal and conjunctival swab was available during the course of the sixth day and revealed a MDR *P. aeruginosa*. Susceptibility testing revealed multiple resistance to ureidopenicillins, cephalosporins, fluoroquinolones, and macrolides. The *Pseudomonas* was only sensitive to meropenem (minimal inhibitory concentration: < 0,25 mg/l) and colistin. Staphylococcus lugdunensis with a resistance to colistin was found in the contact lens case of the same eye. No fungi were detected.

**Table 2:** Antibiotic sensitivity testing of Pseudomonas aeruginosa in the corneal swab and Staphylococcus lugdunensis in the corneal lens case of the right eye; *R* resistant, *I* intermediate, *S* = sensible

Pseudomonas aeruginosa (corneal swab)		Staphylococcus lugdunensis (contact lens case)	
Agent	Antibiotic sensitivity	Agent	Antibiotic sensitivity
Meropenem	S		
Colistin	S	Colistin	R
Erythromycin	R	Erythromycin	S
Azithromycin	R	Azithromycin	S
Ofloxacin	R	Ofloxacin	I
Gentamicin	I	Gentamicin	S
Moxifloxacin	R	Moxifloxacin	S
Piperacillin / tazobactam	I		
Cefotaxime	R		
Ceftriaxone	R		
Ceftazidime	I		
Imipenem	I		
Levofloxacin	I		
Ciprofloxacin	I		

Based on the antibiogram, the use of meropenem and colistin eye drops was considered. Although polymyxin B has structural similarities to colistin, a worsening of the corneal findings was observed during the therapy with Polymyxin B. In addition, the Staphylococcus lugdunensis from the contact lens case was resistant to colistin. Therefore, meropenem eye drops were chosen for further treatment. Meropenem eye drops 50 mg/ml were prepared by the hospital pharmacy. In addition to the local antibiotics, adjunctive systemic meropenem administration was started after the patient was fully informed about the individual curative trial and the off-label use. A subsequent ocular hypertension was successfully treated with a carbonic anhydrase inhibitor (dorzolamide eye drops) and a beta blocker (timolol eye drops). Meropenem eye drops were applied hourly and systemic meropenem was used 1 g three times a day. In addition, systemic voriconazole was given as intravenous infusion (720 mg for the first two infusions and then 480 mg twice daily). This resulted in a rapid improvement over the following days (Fig. 1C: day 7; Fig. 1D: day 15). Additionally topical steroids (dexamethasone) were administered two days after the onset of clinical improvement and systemic therapy was discontinued at the same time after a total administration of 7 days. The patient was discharged on day 20 on topical therapy (dexamethasone 4x/day, gentamicin 4x/day, levofloxacin 4x/day, meropenem 4x/day) with the ability to count fingers (Fig. 1E: day 20). In addition to meropenem eye drops, gentamicin and levofloxacin were continued due to the intermediate susceptibility of P. aeruginosa to these agents and the improvement in corneal status with the combination therapy. The meropenem eye drops continued to be prepared by our hospital pharmacy and provided to the patient during his outpatient treatment. The attempt to discontinue all antibiotics during the course was successful. In total, meropenem eye drops were given for 18 days (hourly for 10 days; every 2 h for one day; 4x/day for 7 days) and systemic meropenem for 7 days (1 g 3x/day). The visual acuity increased continuously. Emergency keratoplasty with a presumably poor prognosis due to extensive neovascularization was avoided.

**Fig. 3:**
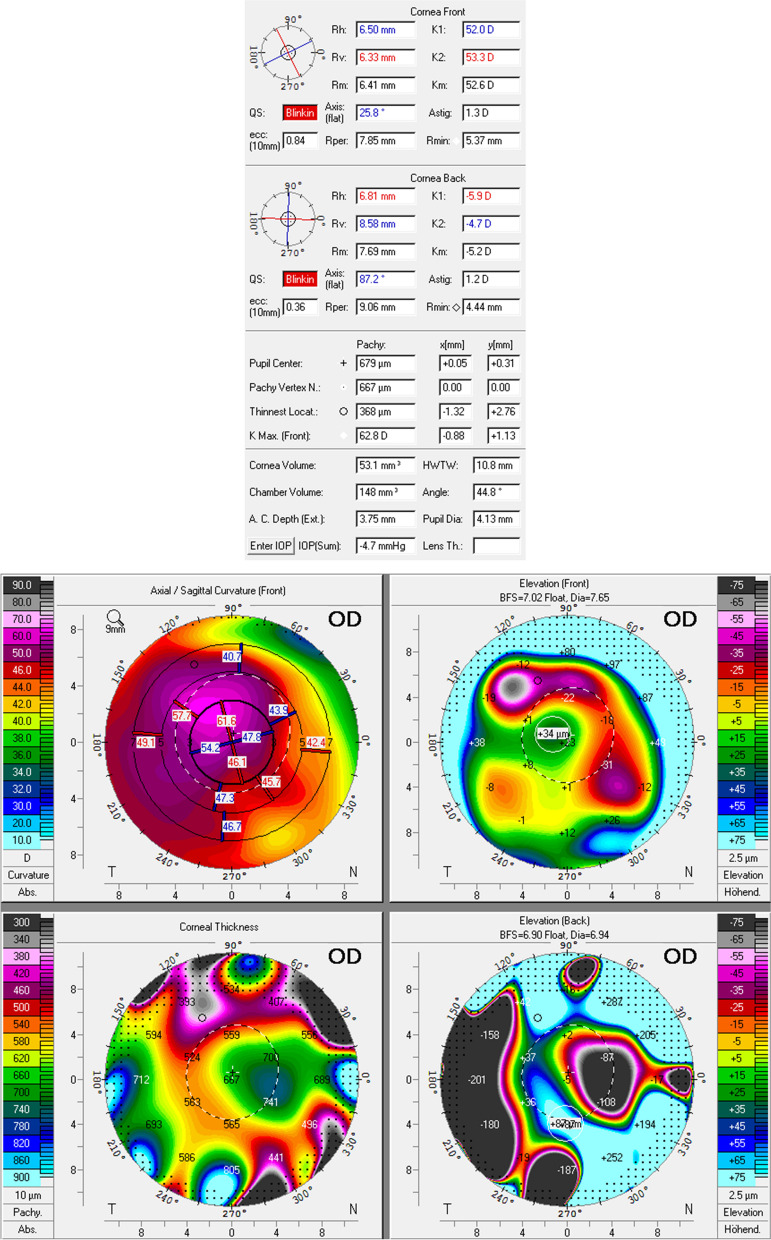
Corneal tomography (Pentacam®) of the right eye after 8 months

After four and eight months, high irregular astigmatism (Fig. 3, follow-up after 8 months) and stromal scarring were evident (Fig. 1F, follow-up after 4 months; Fig. 2B, follow-up after 8 months). Visual acuity with the patient's old glasses was 0.2 (0.70 logMAR) and the intraocular pressure was 18 mmHg in the affected eye. At this point, further therapeutic options would have been a correction with glasses or contact lenses, a deep anterior lamellar keratoplasty (DALK), or a penetrating keratoplasty. In our opinion, the scar was too large for phototherapeutic keratectomy without sufficient residual thickness after laser ablation. Despite these findings and limitations, the patient was satisfied with his current state of his vision. At this time, he preferred not to undergo corneal transplant. He also decided not to get new glasses and stopped wearing contact lenses. After eight months, the patient presented with stable findings and visual acuity. The corneal thickness at the previous thinnest point was 356 µm (Fig. 2B). A follow-up visit is planned to reevaluate visual improvement for example by wearing contact lenses or by surgical options. The option of a corneal transplantation for visual improvement is still available in the future.

## Discussion and conclusion

In our case, *P. aeruginosa* was detected in the corneal and conjunctival swabs and was consistent with the clinical picture. The pathogen was 3MRGN and sensitive to carbapenem β-lactam. Meropenem is a carbapenem β-lactam antibiotic with a broad-spectrum activity against gram-positive and gram-negative bacteria. It is approved by the Food and Drug Administration (FDA) for the treatment of skin, gastrointestinal infections, and bacterial meningitis. Meropenem is potentially effective in ophthalmic use with low minimal inhibitory concentrations against several bacterial subgroups, including *P. aeruginosa *[6]. Meropenem penetrates the cornea well into the anterior chamber [7]. Furthermore, a study demonstrated a satisfactory safety profile for a concentration of 10 mg/ml due to its low toxicity in both existing and disrupted corneal epithelium (to our knowledge, 50 mg/ml has not been studied) [7]. A study showed that meropenem is among the antibiotics to which *P. aeruginosa* is the second most sensitive. *Pseudomonas* is most sensitive to ciprofloxacin, to which the *Pseudomonas*strain in our study was resistant [8]. In experimental models, topical meropenem has been shown to be at least as effective as topical moxifloxacin in treating *P. aeruginosa *keratitis [9]. Systemic meropenem has been used in the therapy of *P. aeruginosa *keratitis [10]. In addition to systemic use, and analogous to the local use of meropenem in the literature, topical meropenem was used in our case [11, 12].

The topical use of meropenem for *P. aeruginosa* corneal ulcers has only been described in a few, previous cases. Pande et al*.* presented the treatment of three nosocomial corneal ulcers caused by *P. aeruginosa*, with meropenem 50 mg/ml being able to save all three eyes [12]. The short stability of meropenem eye drops of 24 h for an opened bottle and 48 h for a closed bottle and the resulting cost of frequent production affects routine use for ulcer treatment.

The increasing resistance of several bacterial pathogens to antibiotics is an ever-growing problem. Therefore, other treatment options have been proposed. For example, the Photo Activated Chromophore for Keratitis (PACK)-Crosslinking is an alternative option to antibiotics, in which the cornea is crosslinked analogous to the treatment of keratoconus. PACK-Crosslinking has been shown to eradicate non-resistant and resistant *P. aeruginosa *[13]. Further studies are needed to fully establish the effect and the potential for use with MDR antibiotic agents. However, in cases of therapy resistance and after other options have been exhausted, an individual curative attempt seems reasonable and may avoid emergency keratoplasty with a poor prognosis.

To summarize, in our case, meropenem eye drops in combination with systemic meropenem were successfully used for contact lens related *P. aeruginosa* corneal ulcer. It is important to refer contact lens wearers with suspected corneal infection to an ophthalmologist for appropriate diagnosis and treatment. For specific antibacterial treatment, it is essential to isolate and identify the bacterial pathogens, determine their antibiotic susceptibility spectrum and start antibiotic treatment as soon as possible according to the antibiogram.

## Data Availability

The datasets used and/or analysed during the current study are available from the corresponding author on reasonable request.

## Abbreviations

FDA: Food and Drug Administration
MDR: Multidrug-resistant
MRGN: Multidrug-resistant gram-negative
PACK-Crosslinking: Photo Activated Chromophore for Keratitis-Crosslinking
P. aeruginosa: Pseudomonas aeruginosa
